# Factors affecting women’s nutritional security in rural Bangladesh: The role of livestock and other socioeconomic characteristics

**DOI:** 10.1371/journal.pone.0335146

**Published:** 2025-11-20

**Authors:** Fatema Tuj Zohora Hira, Mohammad Jahangir Alam, Ismat Ara Begum, Md. Abdullah Al Mamun, Md. Asif Iqbal, Farzana Yeasmin

**Affiliations:** 1 Department of Agribusiness and Marketing, Bangladesh Agricultural University, Mymensingh, Bangladesh; 2 Department of Agricultural Economics, Bangladesh Agricultural University, Mymensingh, Bangladesh; Lusofona University of Humanities and Technologies: Universidade Lusofona de Humanidades e Tecnologias, PORTUGAL

## Abstract

In every household, women play a crucial role in shaping the foundation of food and nutrition. They are primarily responsible for ensuring nutritional security for all members. However, women often find themselves in vulnerable positions within this context. With this in mind, the study aimed to evaluate the nutritional security status of women and explore its determinants. Data were extracted from the Bangladesh Integrated Households Survey-2018. A total of 5604 women were considered, and their nutritional security status was measured based on their minimum dietary diversity intake and sufficient calorie consumption in a 24-hour period. The data were divided into two subsets: households with livestock and households without livestock, to examine the impact of livestock ownership on women’s nutritional security status. The findings revealed that within the overall population, approximately 9% of women have achieved nutritional security. Interestingly, a higher proportion of women, around 11%, from households with livestock were found to be nutritionally secure compared to those without livestock, where only about 7% of women achieved nutritional security. A binary logit regression model was utilized to explore significant predictors and found that livestock ownership, women’s education level, household income, farm size, household size, ownership of a mobile phone by women, and women`s nutritional knowledge were significantly positively associated with their nutritional security status. When examining the subset of women from households without livestock, it was found that their monthly income, farm size, and women’s nutritional knowledge had insignificant impacts compared to women from households with livestock. Addressing the role of livestock, the study concludes that the predictors of women’s nutritional status are not equally significant based on livestock ownership. The study’s findings will assist in designing and formulating future policies and development programs addressing this newly acquired knowledge.

## 1. Introduction

Livestock is a vital sub-sector of agriculture in Bangladesh, significantly contributing to agricultural output and the national economy [1]. The country hosts a diverse range of livestock, primarily including cattle, goats, sheep, poultry (such as chickens and ducks), and an increasing number of buffalo. In the fiscal year 2022−23, the livestock population comprised approximately 24.8 million cattle, 26.9 million goats, 1.5 million buffaloes, and 3.8 million sheep. However, the poultry industry remains predominant, with a population of 385.8 million birds [2]. Smallholder farmers dominate the sector, particularly in cattle and poultry rearing, which are the most economically significant due to their contributions to meat, milk, and egg production. Nationally, Bangladesh produced an estimated 14.1 million metric tons of milk and 8.7 million metric tons of meat, with poultry accounting for nearly 50% of total meat production. Egg production reached approximately 23.4 billion annually [2]. In the fiscal year 2022–23, livestock productivity grew by an estimated 3.2%, contributing about 16.4% to the agricultural GDP and approximately 1.9% to the overall GDP [3].

One in every three persons worldwide faces a malnutrition problem [4], contributing to the death of one in five mothers [5]. Like many other developing countries, Bangladesh grapples with overpopulation and malnutrition as significant concerns [6]. Recognized as key players in nutrition, women`s dietary diversity serves as a major indicator of nutritional outcomes for women and children [7,8]. Dietary diversity denotes the number of different food groups consumed over a specific period, often a day or a week [9,10]. In developing countries, starchy staple foods are predominant, while fruits, vegetables, and animal-source foods are consumed in limited quantities, placing the population, especially women, at high risk of micronutrient deficiency [11–14]. Poor dietary habits and limited access to a diverse range of foods contribute to maternal malnutrition [13,15,16], which has become a significant global health challenge, increasing the risk of adverse pregnancy outcomes, poor infant survival, and heightened susceptibility to chronic diseases later in life [17].

Dietary diversification is recommended to ensure adequate nutrient intake [18,19], as numerous studies have shown that dietary diversity correlates with increased nutrient intake [18,20,21]. Maintaining good nutrition requires adequate nutrient intake, often associated with food variety and diet quality [22]. Among South Asian countries, Bangladesh has the lowest per capita calorie availability and faces frequent adverse climate conditions, which pose risks to domestic agricultural productivity, including food availability [23,24].

Malnutrition poses a significant concern in Bangladesh and other developing countries [6]. Only 14% of individuals in Bangladesh exhibit a higher dietary diversity (DD) score, suggesting potential issues with the country’s overall nutritional quality [25]. Socio-regional disparities in nutritional status persist globally [26], and Bangladesh experiences significant variations in nutritional status across regions [27–29], impacting central budget allocation [30] and socio-cultural perceptions [31]. Differential regional budget allocation exacerbates malnutrition among women and children [32,33]. Socio-cultural disparities, including gender norms and food culture, contribute to the spatial distribution of malnutrition, particularly affecting women and children [34]. Considering their energy expenditure and social status, these factors also impact women’s health [35]. Social and religious norms shape food customs [36]. A healthy diet plays a crucial role in the nutritional status of women of reproductive age, but many women in Bangladesh struggle to achieve dietary diversity [37–40].

Some studies frequently regard dietary diversity as an outcome variable, often measured through the 24-hour recall method [37,41,42]. However, they often overlook sufficient calorie consumption, which ensures women’s nutrient adequacy. Numerous studies have established a connection between dietary diversity and various maternal demographic factors, such as age, parity, education level, nutritional knowledge, employment status, household wealth, size, access to resources, morbidity, and environmental factors like water source, sanitation, and food safety [37,42,43]. Also, this research has highlighted significant gaps between nutrient intakes and requirements, underscoring the importance of dietary diversity in enhancing nutrient absorption and overall health [44,45].

Additionally, several studies have shown that women generally possess a better understanding of nutrition across various domains, as demonstrated by numerous assessments of nutritional knowledge [27]. This knowledge implies that individuals with more diverse diets are likely to meet their nutrient requirements, highlighting the connection between dietary diversity and nutrient intake. Some studies have identified significant gaps between nutrient intakes and requirements, emphasizing the importance of dietary diversity in improving nutrient absorption and overall health [13,41]. These studies have also explored predictors of dietary diversity and its relationship with nutritional status, particularly among pregnant women, including household food consumption scores [46]. Some studies have shown that women engage in livestock activities such as rearing, fodder collection, feeding, healthcare, milking, and household-level processing, which could have both positive and negative impacts on women`s nutrition [47–50]. Thus, it is evident that there is a shortage of studies considering dietary diversity alongside sufficient calorie consumption as indicators of nutritional security for women. While a more diverse diet may lead to higher nutrient intake, ensuring adequate calorie consumption based on age and workload could provide a more direct and comprehensive measure. However, the combination of minimum dietary intake and calorie consumption has received little attention in the context of women’s nutritional security. Therefore, this study aims to address this knowledge gap by assessing women’s nutritional security status and exploring the determinants of that status. Additionally, it provides guidelines for policy formulation and identifies areas for future research, contributing empirical evidence that integrates dietary diversity and calorie adequacy as joint indicators of women’s nutritional security, while explicitly comparing households with and without livestock, an approach that has received limited attention in the Bangladeshi context.

## 2. Methods

### 2.1 Data source

The study utilized data from the Bangladesh Integrated Household Survey (BIHS) 2018, a household survey conducted by Data Analysis and Technical Assistance Limited under the supervision of the International Food Policy Research Institute.

A robust statistical method was employed to calculate the total BIHS sample survey, which comprised 5,604 households distributed across 325 primary sampling units (referred to as villages, the rural areas of Bangladesh). The sample size was determined through two stages: first, by selecting Primary Sampling Units (PSUs), and then subsequently by selecting households within the PSUs [51]. The study analyzed the entire dataset and created a subset that included all households engaged in livestock production. Among the 5,604 households surveyed, 2,725 were involved in livestock production. It is important to note that the observations in the dataset were obtained through a random sampling technique, and no information about livestock-related interventions was present in the dataset.

### 2.2 Description of the study variables

#### Measurement/estimation of different exploratory variables.

Table 1 presents the various independent/ exploratory variables used in the study. Providing a general description of these variables can enhance understanding of their nature and distribution.

**Table 1:** Description of the exploratory variables.

Variable	Nature	Description
Years of schooling of women	Discrete	Represent the number of years of formal schooling received by the women
Household size	Discrete	Represent the total number of persons living in a household
Monthly income	Continuous	The household total income generated or received in a month is estimated in TK (BDT).
Farm size	Continuous	The area (decimal) is managed for cultivation by the household in the current year.
Have livestock	Dichotomous	Whether the household has engaged in livestock production or not.
Employment status	Dichotomous	Whether a woman formally or informally works outside of the home for a salary or not
Own mobile phone	Dichotomous	Whether the women have their mobile phones or not
Nutritional knowledge	Categorical (ordered)	A composite variable was created from several nutrition-related questions.
Session with doctor	Dichotomous	Whether the women had any sessions with a doctor or not

The measure of women`s nutrition knowledge, as referenced in the above table, was developed to assess their understanding of key nutrition concepts relevant to rural Bangladeshi contexts. This aligns with the survey’s comprehensive approach, which includes detailed data collection on individual household members` dietary intake and anthropometric measurements. It was assessed using several nutrition-related questions:

- How long after birth should a baby start breastfeeding?
- What should a woman do with the “first milk” or colostrum?
- How often should a baby breastfeed?
- Do you think that infants under six months of age should be given water if it is hot weather?
- When should a baby receive liquids (including water) besides breast milk?
- At what age should a baby first start to receive food in addition to breastfeeding?
- What should a woman do regarding child feeding when a child under six months has diarrhea?
- What should a woman do regarding child feeding when a child over six months has diarrhea?
- When should you wash your hands?
- Things you can do to encourage young children to eat their food.
- What kind of foods does a young child (<24 months) need to grow and develop their brain?

Each indicator/question mentioned above is binary in response types, indicating whether women know specific information. A response is coded as `1` if a woman answers accurately or is well-informed, and `0` if she does not know the answer. This coding system distinguishes between positive (coded as 1) and negative (coded as 0) responses, following established research methodologies [52,53]. Various aspects of breastfeeding practices and maternal caregiving behaviors were evaluated using specific questionnaire items. For example, the timing of breastfeeding initiation was coded as `1` if a woman breastfed immediately after birth or within one hour of delivery and `0` otherwise. Similarly, the importance of providing colostrum to newborns was assessed, with affirmative responses coded as `1` and negative responses as `0`. Breastfeeding frequency based on the baby’s cues was categorized as `1`, while other responses were coded as `0`. Opinions on offering water to infants under six months in hot weather were recorded as `1` for affirmative responses and `0` otherwise. The survey also captured mothers’ awareness of infants receiving liquids or complementary foods at six months or older, coded accordingly. Furthermore, maternal actions during infant diarrhea episodes, including a continuation of breastfeeding for children under six months, were coded as `1`, and cessation or continuation with ORS/home-prepared solution for children over six months were recorded similarly. Handwashing practices before caregiving tasks were assessed, with affirmative responses coded as `1` and uncertainty or negative responses as `0`. Maternal strategies to encourage healthy eating habits in children were recorded as `1` if adopted and `0` if not. Lastly, maternal knowledge about the nutritional needs of young children (<24 months) for optimal growth and brain development was documented. This classification approach aligns with the survey’s design, aiming to capture meaningful variations in nutrition knowledge that may influence household dietary practices and nutritional outcomes.

To aggregate the responses, the sum of all variables for an individual was divided by 11 (the total number of questions) and multiplied by 100 to transform it into a percentage. The scale was further classified into three groups: poor if the score lies between 0% to 50%, fair if the score lies between 51% to 75%, and good if the score lies above 75%. This method of categorization was used by [54].

### 2.3 Outcome/ explanatory variable

The outcome variable of the study is women’s nutritional security status, estimated using dietary diversity and calorie consumption. Dietary diversity is assessed based on the consumption of nine food groups, as detailed in Table 2.

**Table 2:** Food groups used to estimate women’s dietary diversity score.

Food Groups	Food items
Grains, white roots and tubers	Rice, corn, maize, wheat, sorghum, millet, bread, pasta/noodles, barley or any other grains or foods made from these, white potatoes, yam, or other foods made from roots
Vitamin A-rich fruits and vegetables	pumpkin, carrot, squash or sweet potato, ripe mango, ripe papaya, and 100% fruit juice made from these + other regionally available vitamin A-rich fruits and vegetables
Dark green leafy vegetables	leafy vegetables like jute leaf, amaranth greens, broccoli, kale, spinach, etc.
Meat, poultry and fish	Goose, beef, lamb, goat, chicken, duck, fish, dry fish, birds
Eggs	Eggs from poultry or any other bird
Other fruits and vegetables	tomato, onion, eggplant + wild fruits and other locally available vegetables and other fruits, including 100% fruit juice made from these
Organ meat	gizzard, heart, kidney, liver, stomach
Dairy and dairy products	Milk, cheese, yogurt, or other dairy products
Nuts and seeds	Groundnut/peanut, cashew, walnut, Baobab seeds, chia seeds or foods made from these

Source: [37,55].

Women meeting the minimum dietary intake criterion of consuming at least five or more groups [56] and consuming sufficient calories based on their age and daily activity level [57], are classified as nutritionally secure. The sufficient calorie consumption in the last 24 hours has specific cut-off points depending on age and activity level. For instance, women aged 19–30 years with a sedentary lifestyle require 1800–2000 calories daily, while those with moderate to active lifestyles need 2000–2200 or 2400 calories, respectively [58]. Meeting or exceeding these calorie requirements within a 24-hour period defines sufficient calorie consumption for women. The study assumes all women are moderately active due to regular household chores and other activities (formal employment). Calorie requirements for different age groups are as follows: 1400–1600 calories for ages 4–8, 1600–2000 for ages 9–13, 2000 for ages 14–18, 2000–2200 for ages 19–30, 2000 for ages 31–50, and 1800 for ages 51 to above [58]. Applying the minimum dietary intake and calorie requirements cut-off results in a binary variable: 1 indicates nutritional security when both conditions are met, and 0 indicates nutritional insecurity. The assessment of dietary diversity and calorie consumption was conducted in accordance with FAO recommendations [59,60].

### 2.4 Analytical technique

The study employed two primary types of analysis: descriptive and inferential. In the descriptive analysis, summary statistics such as mean, standard deviation, minimum, and maximum were calculated for all continuous and discrete variables. Frequency and percentage distributions were reported for qualitative variables. Both tabular and graphical representations were utilized. For example, employment status, a qualitative variable with two categories (employed and unemployed), was analysed by reporting the percentage of individuals in each category among the total observations.

#### Binary logit regression model.

The outcome variable in this study is binary. To analyze it, the study opted for the binomial logit model, which is suitable when exploratory variables are dichotomous [61]. The equation form of this model is as follows;


$${\text{Ln}[\text{p}/(\text{1}-\text{p})=\;\beta }_{\text{0}}+\beta_{\text{1}}X$$

Where, p is the probability that the event Y occurs, p(Y = 1)[range = 0–1]

The odds ratio is defined as:

p/(1-p) is the “odds ratio’’[range = 0 to ∞]

Ln[p/(1-p)]: log odds ratio, or “logit”[range = -∞ to +∞]

As the outcome variable of this study is reported in binary form, the chosen econometric model is as follows.

For the entire household, the empirical equation is:


$$\begin{array}{c} Y_{\left(women\;nutritional\;security\right)}=\beta_{\text{0}}+\beta_{\text{1}}X_{\left(Have\;livestock\right)}+\beta_{\text{2}}X_{\left(Year\;of\;schooling\right)}+\beta_{\text{3}}X_{\left(Household\;size\right)}\\ \;\;+\beta_{\text{4}}X_{\left(\text{Log of monthly income}\right)}+\beta_{\text{5}}X_{\left(\text{Log of farm area}\right)}+\beta_{\text{6}}X_{\left(\text{Employment status }\right)}+\beta_{\text{7}}X_{\left(\text{Own mobile phone}\right)}\\ \;\;+\beta_{\text{8}}X_{\left(\text{Nutritional knowledge}\right)}+\beta_{\text{9}}X_{\left(\text{session with doctor}\right)} \end{array}$$

And, to empirically analyse the difference between households that have livestock and those that do not, the following equation is specified:


$$\begin{array}{c} Y_{\left(women\;nutritional\;security\right)}=\beta_{\text{0}}+\beta_{\text{1}}X_{\left(Year\;of\;schooling\right)}+\beta_{\text{2}}X_{\left(Household\;size\right)}+\beta_{\text{3}}X_{\left(\text{Log of monthly income}\right)}\\ \;\;+\beta_{\text{4}}X_{\left(\text{Log of farm area}\right)}+\beta_{\text{5}}X_{\left(\text{Employment status }\right)}+\beta_{\text{6}}X_{\left(\text{Own mobile phone}\right)}+\beta_{\text{7}}X_{\left(\text{Nutritional knowledge}\right)}\\ \;\;+\beta_{\text{8}}X_{\left(\text{session with doctor}\right)} \end{array}$$

The Adjusted odds ratio was used to explain the relationship between the explanatory and outcome variables. All analyses were conducted using STATA v17 software.

### 2.5 Ethical approval

The study was conducted following the Declaration of Helsinki. It used publicly available data from the Bangladesh Integrated Household Survey (BIHS), administered by the International Food Policy Research Institute (IFPRI). The BIHS obtained formal informed consent from all respondents before data collection.

## 3. Results

### 3.1 Descriptive statistics of different exploratory variables

Descriptive statistics for the variables used in the study are presented in Appendix S1 Table. On average, women had approximately 2 years of education, with a standard deviation of 4 years. Educational attainment ranged from 0 to 16 years across the entire dataset and its subsets. The average household size was 4, ranging from 1 to 18 members. Households with livestock tended to be slightly larger, with a maximum of 18 members compared to 17 members in households without livestock.

The average monthly household income was approximately TK (1 US$ = 122 TK [62]) 5,500, with a standard deviation of TK 7251, ranging from TK 0 to TK 125,000. Households with livestock had an average monthly income of TK 300 higher than the entire population, ranging from TK 0 to TK 80,000. In contrast, households without livestock had an average income of TK 5,201.23, with the same income range as the entire population.

The average farm size for all households was nearly 9 decimals, ranging from 0 to 170 decimals. Livestock-rearing households had slightly larger farm sizes, averaging 10 decimals with a maximum of 122 decimals, compared to non-livestock-rearing households with an average of 8 decimals and a range from 0 to 170 decimals.

**Fig 1:**
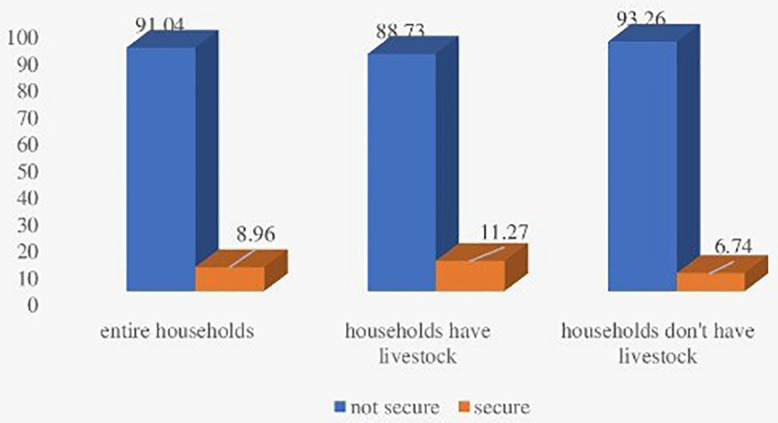
Illustrates notable disparities in the nutritional security status of women based on household livestock ownership. Among women from livestock-owning households, 11.27% were classified as nutritionally secure, compared to only 6.74% in households without livestock. This suggests a positive association between livestock ownership and improved nutritional security for women. When considering the overall population, 8.96% of women were identified as nutritionally secure, indicating that livestock ownership may be a contributing factor to enhanced nutritional outcomes.

Approximately 49% of households reported owning livestock. Employment rates were higher in households with livestock, with 95% of women employed, compared to 85% in all households. Conversely, non-livestock-rearing households had a lower employment rate of 74%. Regarding mobile phone ownership, nearly half of all respondents did not have a personal mobile phone, with a slightly higher percentage of mobile phone users in households without livestock. Regarding nutritional knowledge and health consultations, around 93% of women had poor to fair nutritional knowledge, and 88% did not consult with a doctor. Women’s nutritional knowledge is a composite variable, with all constituent variables illustrated in Fig 1. Across the entire population, approximately 52% of women were aware of the recommended time for initiating breastfeeding after birth. This proportion decreased by 2% and 3% among women from households owning and not owning livestock, respectively.

Regarding handling first milk, approximately 95% of women in both groups know the proper procedure, while nearly 94% of women in households without livestock are aware of it. Almost 50% of women in all groups are knowledgeable about the frequency of breastfeeding for infants. Concerning the provision of water to infants under six months in hot weather, around half of the women across all groups believe that water should not be given. Approximately 95% of women in the entire population, as well as those from households without livestock, are aware of the appropriate age for introducing liquids other than breast milk. This proportion drops slightly to 94% among women from households with livestock. Additionally, about 97% of women across all groups know the recommended age for introducing solid foods to infants. Within group 1, nearly 96% of women are unaware of the appropriate feeding practice for infants under six months suffering from diarrhea, with a slight decrease to 94% among women from households without livestock. Conversely, approximately 87% of women in all groups are knowledgeable about the appropriate diet for children over six months during episodes of diarrhea.

All women in both groups are aware of when to wash their hands. Approximately 89% of women across all groups understand how to encourage their young children to eat their food. Finally, nearly 99% of women in the entire population and households owning livestock are knowledgeable about the foods needed for a young child’s growth and brain development (under 24 months) (Table 3).

**Table 3:** Nutrition knowledge-related indicators of women.

Variables	entire population		household have livestock		household does not have livestock	
	don’t know	know	don’t know	know	don’t know	know
How long after birth should a baby start breastfeeding	48.26	51.74	49.65	50.35	46.92	53.08
What should a mother do with the “first milk” or colostrum	5.68	94.32	4.96	95.04	6.38	93.62
How often should a baby breastfeed	44.66	55.34	43.74	56.26	45.56	54.44
Do you think that infants under 6 months of age should be given water if it is hot weather	47.68	52.32	47.52	52.48	47.84	52.16
When should a baby receive liquids (including water) other than breast milk	4.99	95.01	5.91	94.09	4.1	95.9
At what age should a baby first start to receive food in addition to breastfeeding	2.55	97.45	2.6	97.4	2.51	97.49
What should a mother do regarding child feeding when a child aged less than 6 months has diarrhea	95.71	4.29	97.4	2.6	94.08	5.92
What should a mother do regarding child feeding when a child aged more than 6 months has diarrhea	13.11	86.89	13	87	13.21	86.79
When should you wash your hands	0	100	0	100	0	100
Things you can do to encourage young children to eat their food	11.14	88.86	11.58	88.42	10.71	89.29
What foods does a young child (<24 months) need to grow and develop their brain	0.93	99.07	0.71	99.29	1.14	98.86

### 3.2 Women’s nutritional security status

The results also highlight that the majority of women across all household categories continue to experience nutritional insecurity, with the highest prevalence observed in households without livestock (93.26%). These findings underscore the urgent need for targeted interventions to improve nutritional outcomes, especially in households lacking adequate livestock. At the same time, broader systematic factors affecting women`s nutritional security must also be addressed.

### 3.3 Determinants of women’s nutritional security status

Table 4 presents the results of the binary logit regression model on the nutritional security status of women. The analysis shows that households owning livestock are 1.507 times (p < 0.01) more likely to be nutritionally secure compared to those without livestock. Additionally, for every additional year of formal education attained by women, the likelihood of nutritional security increases by 1.026 times (p < 0.05) in Group 1 and by 1.051 times (p < 0.01) in Group 3. Moreover, each additional household member corresponds to a 1.224 times higher likelihood of nutrition security in Group 1, 1.194 times (p < 0.01) in Group 2, and 1.283 times (p < 0.01) in Group 3. A 1% increase in monthly income is associated with 1.019 times (p < 0.1) higher likelihood of nutritional security in Group 1 and 1.035 times (p < 0.05) in Group 2.

**Table 4:** Determinants of women’s nutrition security (Binary logit regression model).

Women’s nutritional security status (base = no)	Group 1: Entire households (n = 5604)				Group 2: Households have livestock (n = 2725)				Group 3: Households don’t have livestock (n = 2879)			
	OR.	Robust St. Err.	95% Conf Interval		OR.	Robust St. Err.	95% Conf Interval		OR.	Robust St. Err.	95% Conf Interval	
Have livestock (yes)	1.507***	0.156	1.230	1.846								
Years of education for women	1.026**	0.013	1.001	1.051	1.007	0.017	0.975	1.041	1.051***	0.019	1.014	1.089
Household size	1.224***	0.029	1.169	1.281	1.194***	0.035	1.127	1.265	1.283***	0.049	1.191	1.382
Log of monthly income	1.019*	0.010	0.999	1.039	1.035**	0.016	1.004	1.068	1.005	0.014	0.978	1.032
Log of farm area	1.066*	0.036	0.998	1.139	1.079*	0.046	0.992	1.174	1.040	0.056	0.937	1.155
Employment status (Yes)	1.193	0.186	0.879	1.619	1.260	0.407	0.669	2.372	1.187	0.214	0.834	1.689
Own mobile phone (Yes)	1.358***	0.132	1.122	1.644	1.352**	0.166	1.063	1.720	1.365*	0.219	0.996	1.870
Nutritional knowledge (good)	1.338*	0.226	0.960	1.864	1.433*	0.312	0.936	2.194	1.182	0.324	0.691	2.024
Session with doctor (Yes)	1.161	0.161	0.884	1.523	1.120	0.196	0.794	1.580	1.222	0.273	0.789	1.892
Constant	0.017***	0.003	0.012	0.025	0.025***	0.009	0.012	0.052	0.015***	0.004	0.009	0.025
Chi-square (model fit)	157.055***				65.625***				64.69***			
Prob > chi2	0.000				0.000				0.000			
Pseudo r-squared	0.041				0.029				0.038			
Correctly classified (classification test)	91.11%				88.84%				93.23%			
Chi-square (Goodness of fit)	4990.15				2462.76				2531.43			
Probability > chi-square	0.866				0.785				0.726			

Note: *** p < .01, ** p < .05, * p < .1; OR = Adjusted Odds ratio; St. Err. = Standard Error; Conf = Confidence.

Similarly, a 1% increase in farm area corresponds to 1.066 times (p < 0.1) higher likelihood of nutritional security in Group 1 and 1.079 times (p < 0.1) in Group 2. Women who own mobile phones are 1.358 times (p < 0.01), 1.352 times (p < 0.05), and 1.365 times (p < 0.1) more likely to be nutritionally secure in entire households (Group 1), households with livestock (Group 2), and households without livestock (Group 3), respectively. Furthermore, women with good nutritional knowledge are 1.338 times (p < 0.1) (Group 1) and 1.433 times (p < 0.1) (Group 2) more likely to be nutritionally secure than those with poor to fair nutritional knowledge.

## 4. Discussions

The study revealed that approximately 11% of women are considered nutritionally secure. This finding is surprising given the overall progress observed in various sectors within the country, including health and nutrition [63]. Despite these advancements, the proportion of nutritionally secure women remains relatively low, highlighting persistent concerns. Several factors could contribute to this poor nutritional security status. For instance, the study considered a rough estimation of dietary intake and calorie consumption to evaluate estimating nutritional security status. Previous studies have indicated that high dietary diversity is not commonly observed among women in Bangladesh [37,42]. This suggests that many women may not consume adequate food according to their requirements, leading to a lower proportion of nutritionally secure women in the country.

Upon investigating the significant factors influencing women’s nutritional status, it became evident that households engaged in livestock production or possessing livestock appears to significantly enhance women’s nutritional well-being. Livestock serve not only as assets but also as sources of income and food supply, encompassing both large and small animals commonly reared in rural Bangladesh [64]. Over recent decades, the number of larger livestock has increased, contributing noticeably to Gross Domestic Product (GDP) and employment generation [65]. Additionally, milk and meat from livestock serve as valuable sources of protein [66,67]. Cattle and buffalo within households combine food and income sources, potentially reducing nutritional deprivation and enhancing employment opportunities. Livestock production presents a significant opportunity for poverty reduction [68], particularly in the developing world, where it is utilized as a tool for poverty alleviation [69]. As milk and meat consumption continue to increase over time [70] so does the demand, thereby generating employment opportunities [71]. In countries like India, the livestock sector has been targeted to reduce rural poverty [72], with livestock playing a crucial role in nutrition, health security, and employment [73]. These factors collectively underscore the vital role of livestock production in ensuring the nutritional security of women in rural areas of developing countries.

The study identified that women’s years of schooling significantly contribute to their nutritional security status. Formal education enhances individuals’ knowledge about diet, health, and related factors. With a broader knowledge base, women are more likely to understand their dietary needs, appreciate the importance of balanced nutrition, and take better care of their health and that of their family members. Educated woman are typically better equipped to utilize available resources effectively and make informed decisions regarding their health [74]. Higher levels of education are also associated with a better understanding of appropriate feeding practices [75]. Evidence from Bangladesh supports this notion, demonstrating a significant and positive association between higher levels of education and maternal dietary diversity [39]. Furthermore, increased dietary intake is linked to greater nutritional security for women. Therefore, investigating women`s education not only empowers them with knowledge but also contributes to improving their nutritional well-being.

Women from larger families often shoulder more responsibilities compared to those from smaller families. The distribution of household chores and caregiving tasks involves the participation of multiple family members rather than falling solely on one individual, such as the household head. In larger families, women typically engage in a range of activities, including household chores, child care, cooking, tending to livestock and poultry, farm and market visits, and hospital visits for themselves and family members. Conversely, women from small families also undertake similar tasks, but often with limited autonomy, as their roles are shaped by the influence of their husbands or household heads. By contrast, women in larger families tend to experience a shift in household dynamics, often gaining greater decision-making power and more flexibility in managing both domestic and productive activities. They gain increased access and control over resources and participate in household and farming decisions, which empowers them. The presence of more family members in larger households bolsters women’s confidence levels [76]. Moreover, larger households tend to have higher total earnings compared to smaller households, enabling them to afford a wider variety of foods. For example, households with dual incomes are more likely to achieve better food security scores than single-income households where both spouses are employed. Previous studies have shown a positive correlation between household size and women’s nutritional intake [46].

The monthly income of a family significantly influences the nutritional security status of women. An increase in income often correlates with greater dietary diversity. Higher-income individuals generally have greater purchasing power, allowing them to allocate more funds toward food products. This results in increased consumption of a variety of foods and ensures adequate nutrient intake. In addition to purchasing food, higher income levels also facilitate investment in nutritional education and knowledge [77]. Income can have multifaceted effects on various aspects of life, but it consistently leads to increased expenditure on food items, thereby enhancing the nutritional security of all household members, including women.

The farm area serves as both a source of food production and income generation for households. It not only provides food for consumption but also generates revenue through the sale of produce or land rental. Research indicates that a larger farm area tends to positively impact women’s nutritional security status. Contrary to these findings, a positive relationship between food intake and farm size, indicating that larger farm owners tend to consume more food [78]. Moreover, in Bangladesh, where large farm owners typically have higher incomes, increased food intake has been observed. Another study found that larger farm-size holders were more likely to achieve food security compared to smallholders in Bangladesh [79]. Therefore, a larger farm area contributes positively to women’s nutritional security status.

Mobile phones have become ubiquitous and indispensable in the contemporary era, with at least one device in every household. The possession of mobile phones by women can significantly enhance their nutritional security status. These devices enable women to communicate as needed and stay updated with information about their surroundings. Moreover, mobile phones provide a convenient platform for accessing media, through which women can obtain crucial information on food and nutritional security. Media exposure allows women to acquire knowledge about nutrition, including healthy recipes, dietary guidelines, and nutritional content [80,81]. Additionally, exposure to media positively influences women`s nutritional behaviors [81]. Furthermore, mobile phones facilitate the use of applications for weight and fitness measurements, as well as promote social support and peer learning on nutritional aspects [80,81]. In many rural Bangladeshi households, particularly those involved in livestock rearing, women often serve as the primary caretakers but may lack the autonomy to own or use mobile phones, highlighting intra-household inequality [82]. In rural areas, the primary barriers to mobile phone adoption are rooted in broader socioeconomic constraints, rather than being attributable solely to livestock ownership. Factors such as illiteracy, lack of access to electricity, and low household wealth were identified as significant demographic constraints to mobile phone ownership [83]. Thus, with access to a mobile phone, women can actively enhance their nutritional security status by leveraging communication, information, and support networks provided by these devices.

It is crucial to emphasize that the observed differences in nutritional security result from the interplay of multiple determinants rather than livestock ownership alone. While livestock provides both food and income opportunities, its nutritional benefits are enhanced when women are educated, households have higher incomes, and mobile phone access facilitates nutritional awareness. Larger households may also improve food security by pooling resources, and farm size contributes to both food availability and income. Additionally, nutritional knowledge enables women to translate resources into healthier dietary practices. These findings underline that livestock is one component within a complex system influencing women’s nutritional security.

A solid understanding of nutrition among women plays a crucial role in elevating their nutritional security status. This knowledge not only influences a woman`s dietary diversity but also benefits all household members. Nutrition knowledge encompasses a breadth of information about dietary needs, which, when internalized, positively shapes attitudes and translates into real-life practices. Proficient nutritional knowledge empowers women to make informed dietary choices, heightens awareness of nutritional requirements, improves resource management, and equips them with strategies to address malnutrition [84,85]. These findings are consistent with a study conducted in Kenya, emphasizing the universal significance of women`s nutrition knowledge in enhancing household nutritional security [41].

## 5. Conclusions and recommendations

Women’s nutritional security in rural Bangladesh remains inadequate, reflecting both economic and social vulnerabilities. This study demonstrates that livestock ownership is a significant determinant of women’s nutritional security, providing both food and economic stability. Although the difference in nutritional security between households with and without livestock may not be large, it is nonetheless statistically significant. Livestock not only contributes directly to women`s nutritional security but also holds a prestigious position as a valuable in rural areas. At the same time, other factors beyond livestock ownership also play important roles and should not be overlooked. These findings underscore that women’s nutritional security is shaped by the interaction of household-level resources and individual-level capabilities.

Based on the findings, several recommendations emerge. To enhance livestock production and improve women`s nutritional outcomes, comprehensive programs and policies should focus on cattle production and its key determinants, supported by sustainable and profitability infrastructure. Both formal and informal education, along with community awareness campaigns, should be promoted to foster a culture of learning. Leveraging contemporary technologies, particularly mobile phones, can facilitate knowledge dissemination, behavioral change and nutrition awareness among women. Interventions should prioritize regions with low livestock ownership, where improved access can substantially enhance women’s nutritional security.

Furthermore, a multifaceted policy approach that addresses the key factors identified in this study is likely to achieve better nutritional outcomes than a single or one-dimensional policy approach. Prioritizing women`s nutritional security at the national level is particularly recommended.

To translate the study findings into practice, both governmental and non-governmental organizations play crucial roles and should incorporate these insights into policy planning and development projects aimed at enhancing women`s nutritional status.

## 6. Limitations

The study explores new avenues of knowledge, but it also has its limitations. Firstly, it relies on cross-sectional data and uses the 24-hour recall method to estimate dietary diversity scores, which may not fully capture the dietary habits of the women accurately. Secondly, the dataset does not include comprehensive data on livestock outputs, such as milk, eggs, and meat consumption, which makes disaggregated analysis impossible. This represents a limitation of the study and could be addressed in future research. Additionally, assuming all women to be moderately active when estimating their calorie consumption might not reflect the diversity in activity levels among women. To improve future studies, it is recommended to consider employing time series analysis or longer recall periods (such as 7 or 15 days) to assess women`s dietary diversity. This approach, along with more precise calorie consumption estimations based on age and activity levels, could yield more robust outcomes. Unfortunately, the study was limited to using only cross-sectional data due to constraints, thus missing out on the longitudinal perspectives that panel data can provide.

In future research, exploring additional predictors beyond those examined in the current study would be beneficial. Different socio-economic contexts may uncover new factors influencing women`s nutritional security status. This expansion of predictors could enhance the comprehensiveness and applicability of future research findings.

## Supporting information

S1 Table Descriptive Statistics of different independent variables. (DOCX)

## References

[1] Sarma PK. Participation in livestock-based interventions and its impact on food security in Bangladesh: A quasi-experimental method. Cleaner and Circular Bioeconomy. 2024;9:100098. doi: 10.1016/j.clcb.2024.100098

[2] BBS (Bangladesh Bureau of Statistics), Yearbook of Agricultural Statistics, (2022). Link: 2024-12-09-02-46-abf36d3293484bb2e9e883e82e8a1efc.pdf

[3] Ahmed A, Bakhtiar MM, Mahzab MM. Food security and nutrition in Bangladesh: Evidence-based strategies for advancement. International Food Policy Research Institute. 2024.

[4] International Food Policy Research Institute (IFPRI), Bangladesh Integrated Household Survey (BIHS) 2018-2019. (2020). doi: 10.7910/DVN/NXKLZJ

[5] Middleton P, Crowther C, Bubner T, Flenady V, Bhutta Z, Son Trach T, et al. PROTOCOL: Nutrition interventions and programs for reducing mortality and morbidity in pregnant and lactating women and women of reproductive age: a systematic review. Campbell Systematic Reviews. 2012;8(1):1–52. doi: 10.1002/cl2.84

[6] Khandoker S, Singh A. Women’s decision making autonomy in household and its effect on dietary diversity: evidence from nationally representative panel data of Bangladesh. In: 2021.

[7] Arimond M, Ruel MT. Dietary diversity is associated with child nutritional status: evidence from 11 demographic and health surveys. J Nutr. 2004;134(10):2579–85. doi: 10.1093/jn/134.10.2579 15465751

[8] Rah JH, Akhter N, Semba RD, de Pee S, Bloem MW, Campbell AA, et al. Low dietary diversity is a predictor of child stunting in rural Bangladesh. Eur J Clin Nutr. 2010;64(12):1393–8. doi: 10.1038/ejcn.2010.171 20842167

[9] WFP. January 2009. 10.1016/s0169-8141(08)00187-x

[10] FAO. Guidelines for measuring household and individual dietary diversity, 2010. doi: 613.2KEN

[11] Arimond M, Elin L, Wiesmann D, Joseph M, Carriquiry A. Dietary diversity as a measure of women’s diet quality in resource-poor areas: Results from rural Bangladesh site. Food and Nutrition Technical Assistance. 2008;58.

[12] USAID. Maternal dietary diversity and the implications for children’s diets in the context of food security. 2012.

[13] Arimond M, Wiesmann D, Becquey E, Carriquiry A, Daniels MC, Deitchler M, et al. Simple food group diversity indicators predict micronutrient adequacy of women’s diets in 5 diverse, resource-poor settings. J Nutr. 2010;140(11):2059S-69S. doi: 10.3945/jn.110.123414 20881077 PMC2955880

[14] Amugsi DA, Lartey A, Kimani E, Mberu BU. Women’s participation in household decision-making and higher dietary diversity: findings from nationally representative data from Ghana. J Health Popul Nutr. 2016;35(1):16. doi: 10.1186/s41043-016-0053-1 27245827 PMC5026004

[15] Moursi MM, Arimond M, Dewey KG, Trèche S, Ruel MT, Delpeuch F. Dietary diversity is a good predictor of the micronutrient density of the diet of 6- to 23-month-old children in Madagascar. J Nutr. 2008;138(12):2448–53. doi: 10.3945/jn.108.093971 19022971

[16] Headey D, Ecker O. Rethinking the measurement of food security: from first principles to best practice. Food Sec. 2013;5(3):327–43. doi: 10.1007/s12571-013-0253-0

[17] Melku M, Addis Z, Alem M, Enawgaw B. Prevalence and Predictors of Maternal Anemia during Pregnancy in Gondar, Northwest Ethiopia: An Institutional Based Cross-Sectional Study. Anemia. 2014;2014:108593. doi: 10.1155/2014/108593 24669317 PMC3942101

[18] Lee SE, Talegawkar SA, Merialdi M, Caulfield LE. Dietary intakes of women during pregnancy in low- and middle-income countries. Public Health Nutr. 2013;16(8):1340–53. doi: 10.1017/S1368980012004417 23046556 PMC10271363

[19] Becquey E, Capon G, Martin-Prevel Y. Validation of dietary diversity as a measure of the micronutrient adequacy of women’s diets: results from Ouagadougou (Burkina Faso). 2009.

[20] Jayawardena R, Byrne NM, Soares MJ, Katulanda P, Yadav B, Hills AP. High dietary diversity is associated with obesity in Sri Lankan adults: an evaluation of three dietary scores. BMC Public Health. 2013;13:314. doi: 10.1186/1471-2458-13-314 23566236 PMC3626879

[21] Black MM. Animal source foods to improve micronutrient nutrition and human function in developing countries: micronutrient deficiencies and cognitive functioning. Journal of Nutrition. 2003;133:3927S-3931S. 10.1093/jn/133.11.3927S PMC3140638 14672291

[22] Zainal Badari SA, Arcot J, Haron SA, Paim L, Sulaiman N, Masud J. Food variety and dietary diversity scores to understand the food-intake pattern among selected Malaysian households. Ecol Food Nutr. 2012;51(4):265–99. doi: 10.1080/03670244.2012.674445 22794127

[23] Ruane AC, Major DC, Yu WH, Alam M, Hussain SkG, Khan AS, et al. Multi-factor impact analysis of agricultural production in Bangladesh with climate change. Global Environmental Change. 2013;23(1):338–50. doi: 10.1016/j.gloenvcha.2012.09.001

[24] Ericksen P, Thornton P, Notenbaert A. Mapping hotspots of climate change and food insecurity in the global tropics: Appendix 1 SOFI Country Group Composition. 2011.

[25] Shourove JH, Meem FC, Rahman M, Islam GMR. Is women’s household decision-making autonomy associated with their higher dietary diversity in Bangladesh? Evidence from nationally representative survey. PLOS Glob Public Health. 2023;3(7):e0001617. doi: 10.1371/journal.pgph.0001617 37467185 PMC10355378

[26] Schwekendiek D. Height and weight differences between North and South Korea. J Biosoc Sci. 2009;41(1):51–5. doi: 10.1017/S002193200800299X 18647440

[27] Arganini C, Saba A, Comitato R, Virgili F, Turrini A. Gender Differences in Food Choice and Dietary Intake in Modern Western Societies. Public Health - Social and Behavioral Health. InTech. 2012. doi: 10.5772/37886

[28] Gupta S, Sunder N, Pingali PL. Market Access, Production Diversity, and Diet Diversity: Evidence From India. Food Nutr Bull. 2020;41(2):167–85. doi: 10.1177/0379572120920061 32522130

[29] Jones AD. On-Farm Crop Species Richness Is Associated with Household Diet Diversity and Quality in Subsistence- and Market-Oriented Farming Households in Malawi. J Nutr. 2017;147(1):86–96. doi: 10.3945/jn.116.235879 27733520

[30] Mithun MdMZ. Regional development planning and disparity in Bangladesh. EJBME. 2021;11(1):010–20. doi: 10.18685/ejmr(8)1_ejbme-20-013

[31] Khan MdS, Haque S, Sarkar MAR, Hoque MdN, Noman SMMH, Wahid T. Thinking out of the ‘Man box’: An intersectional exploration of gender dynamics in northern Bangladesh via gender tracking framework. World Development Sustainability. 2023;3:100100. doi: 10.1016/j.wds.2023.100100

[32] Akseer N, Al-Gashm S, Mehta S, Mokdad A, Bhutta ZA. Global and regional trends in the nutritional status of young people: a critical and neglected age group. Ann N Y Acad Sci. 2017;1393(1):3–20. doi: 10.1111/nyas.13336 28436100

[33] Carlson GJ, Kordas K, Murray-Kolb LE. Associations between women’s autonomy and child nutritional status: a review of the literature. Matern Child Nutr. 2015;11(4):452–82. doi: 10.1111/mcn.12113 24521434 PMC6860340

[34] Monterrosa EC, Frongillo EA, Drewnowski A, de Pee S, Vandevijvere S. Sociocultural Influences on Food Choices and Implications for Sustainable Healthy Diets. Food Nutr Bull. 2020;41(2_suppl):59S-73S. doi: 10.1177/0379572120975874 33356592

[35] Ruel MT, Alderman H, Maternal and Child Nutrition Study Group. Nutrition-sensitive interventions and programmes: how can they help to accelerate progress in improving maternal and child nutrition?. Lancet. 2013;382(9891):536–51. doi: 10.1016/S0140-6736(13)60843-0 23746780

[36] Abou-Rizk J, Jeremias T, Cocuz G, Nasreddine L, Jomaa L, Hwalla N, et al. Food insecurity, low dietary diversity and poor mental health among Syrian refugee mothers living in vulnerable areas of Greater Beirut, Lebanon. Br J Nutr. 2022;128(9):1832–47. doi: 10.1017/S0007114521004724 34842129 PMC9592946

[37] Haque S, Salman M, Rahman MS, Rahim ATMA, Hoque MN. Mothers’ dietary diversity and associated factors in megacity Dhaka, Bangladesh. Heliyon. 2023;9(8):e19117. doi: 10.1016/j.heliyon.2023.e19117 37636472 PMC10450986

[38] Haque S, Salman M, Hossain MS, Saha SM, Farquhar S, Hoque MN, et al. Factors associated with child and maternal dietary diversity in the urban areas of Bangladesh. Food Sci Nutr. 2023;12(1):419–29. doi: 10.1002/fsn3.3755 38268877 PMC10804084

[39] Nguyen PH, Avula R, Ruel MT, Saha KK, Ali D, Tran LM, et al. Maternal and child dietary diversity are associated in Bangladesh, Vietnam, and Ethiopia. J Nutr. 2013;143(7):1176–83. doi: 10.3945/jn.112.172247 23658424

[40] Roy BD. Milk: the new sports drink? A Review. J Int Soc Sports Nutr. 2008;5:15. doi: 10.1186/1550-2783-5-15 18831752 PMC2569005

[41] Ekesa BN, Walingo MK, Onyango MA. Dietary diversity, nutrition status and morbidity of pre-school children in Matungu division, Western Kenya. IJFSNPH. 2009;2(2):131. doi: 10.1504/ijfsnph.2009.029279

[42] Haque S, Salman M, Hossain MS, Saha SM, Farquhar S, Hoque MN, et al. Factors associated with child and maternal dietary diversity in the urban areas of Bangladesh. Food Sci Nutr. 2023;12(1):419–29. doi: 10.1002/fsn3.3755 38268877 PMC10804084

[43] Abebe H, Alemteshay T. Determinants of Dietary Diversity Consumption and Nutritional Status of Pregnant Women Attending Armed Forces Comprehensive Specialized Hospital, Addis Ababa, Ethiopia. Food Proc Nutr Sci. 2020;1(1):51–79. doi: 10.46619/fpns.2020.1-1005

[44] Arimond M, Wiesmann D, Becquey E, Carriquiry A, Daniels MC, Deitchler M, et al. Simple food group diversity indicators predict micronutrient adequacy of women’s diets in 5 diverse, resource-poor settings. J Nutr. 2010;140(11):2059S-69S. doi: 10.3945/jn.110.123414 20881077 PMC2955880

[45] Ekesa BN, Walingo MK, Onyango MA. Dietary diversity, nutrition status and morbidity of pre-school children in Matungu division, Western Kenya. IJFSNPH. 2009;2(2):131. doi: 10.1504/ijfsnph.2009.029279

[46] Saaka M, Mutaru S, Osman SM. Determinants of dietary diversity and its relationship with the nutritional status of pregnant women. J Nutr Sci. 2021;10:e14. doi: 10.1017/jns.2021.6 33889397 PMC8057399

[47] Amin H, Ali T, Ahmad M, Zafar MI. Gender and Development: Roles of Rural Women in Livestock Production in Pakistan. Pakistan Journal of Agricultural Sciences. 2010;47(1).

[48] Herrero M, Grace D, Njuki J, Johnson N, Enahoro D, Silvestri S, et al. The roles of livestock in developing countries. Animal. 2013;7 Suppl 1:3–18. doi: 10.1017/S1751731112001954 23121696

[49] Naz S, Khan NP, Afsar N, Shah AA. Women’s Participation and Constraints in Livestock Management: A Case of Khyber Pakhtunkhwa Province Pakistan. SJA. 2018;34(4). doi: 10.17582/journal.sja/2018/34.4.917.923

[50] Saini V, Saini R. Livestock sector: A tool for women empowerment. The Pharma Innovation Journal. 2021;10(1):139–43.

[51] International Food Policy Research Institute (IFPRI). Bangladesh Integrated Household Survey (BIHS) 2018-2019. 2020. doi: 10.7910/DVN/NXKLZJ

[52] Haque S, Al Rafi DA, Zaman N, Salman M, Al Noman MA, Hoque MN, et al. Nutritional status of under-five aged children of ready-made garment workers in Bangladesh: A cross-sectional study. PLoS One. 2023;18(4):e0284325. doi: 10.1371/journal.pone.0284325 37053193 PMC10101446

[53] S H, M S, DAA R, MAA N, MN H, SA S, et al. Providing Antenatal Care Facility is the Most Effective Way to Improve Nutritional Knowledge of Mothers Working in the Ready-Made Garment Industry of Bangladesh. JAFE. 2022;03(01). doi: 10.47440/jafe.2022.3105

[54] Armar-Klemesu M, Ruel MT, Maxwell DG, Levin CE, Morris SS. Poor maternal schooling is the main constraint to good child care practices in Accra. J Nutr. 2000;130(6):1597–607. doi: 10.1093/jn/130.6.1597 10827216

[55] FAO, Minimum dietary diversity for women, FAO. 2016. 10.4060/cb3434en

[56] FAO. Minimum Dietary Diversity for Women- A Guide to Measurement, Food and Agriculture Organization of the United Nations and USAID’s Food and Nutrition Technical Assistance III Project (FANTA), managed by FHI, 2016.

[57] Manikas I, Ali BM, Sundarakani B. A systematic literature review of indicators measuring food security. Agric Food Secur. 2023;12(1):10. doi: 10.1186/s40066-023-00415-7 37193360 PMC10161169

[58] Zelman KM, Braverman J. How Many Calories Do You Really Need? WebMD.20243. [cited August 10, 2024]. https://www.webmd.com/diet/calories-chart

[59] Shaheen N, Rahim ATMA, Mohiduzzaman M, Banu CP, Bari LM, Tukun AB, et al. Food composition table for Bangladesh. 2013. https://www.fao.org/fileadmin/templates/food_composition/documents/FCT_10_2_14_final_version.pdf

[60] FAO. Guidelines for measuring household and individual dietary diversity. Food and Agriculture Organization of the United Nations. 2011. https://www.fao.org/fileadmin/user_upload/wa_workshop/docs/FAO-guidelines-dietary-diversity2011.pdf

[61] Long JS, Freese J. Regression Models for Categorical Dependent Variables Using STATA, 2001. http://www.amazon.com/dp/1597180114

[62] Bangladesh Bank, Bangladesh Bank. 2025. [cited June 18, 2025]. https://www.bb.org.bd/en/index.php/econdata/exchangerate

[63] BER, Bangladesh-Economic-Review-2023 - Finance Division, Ministry of Finance-Government of the People\’s Republic of Bangladesh. 2023. [cited April 28, 2024. https://mof.portal.gov.bd/site/page/28ba57f5-59ff-4426-970a-bf014242179e/Bangladesh-Economic-Review-2023

[64] Islam MA, Khatun MM, Werre SR, Sriranganathan N, Boyle SM. A review of Brucella seroprevalence among humans and animals in Bangladesh with special emphasis on epidemiology, risk factors and control opportunities. Vet Microbiol. 2013;166(3–4):317–26. doi: 10.1016/j.vetmic.2013.06.014 23867082

[65] Salim H. Livestock Economy at a glance (2020-2021), Bangladesh Bureau of Statistics. 2021. http://www.dls.gov.bd/site/page/22b1143b-9323-44f8-bfd8-647087828c9b/Livestock-Economy

[66] Roy BD. Milk: the new sports drink? A Review. J Int Soc Sports Nutr. 2008;5:15. doi: 10.1186/1550-2783-5-15 18831752 PMC2569005

[67] Kumar P, Chatli MK, Mehta N, Singh P, Malav OP, Verma AK. Meat analogues: Health promising sustainable meat substitutes. Crit Rev Food Sci Nutr. 2017;57(5):923–32. doi: 10.1080/10408398.2014.939739 25898027

[68] Peden D, Tadesse G, Mammo M. Improving the water productivity of livestock: an opportunity for poverty reduction. IN:, Integrated Water and Land Management Research and Capacity Building Priorities for Ethiopia. (2003) 57–65. on 23/08/2016. http://mahider.ilri.org/handle/10568/274

[69] Thornton PK, Kruska RL, Henninger N, Kristjanson PM, Reid RS, Atieno F, et al. Mapping poverty and livestock in the developing world. Health. 2002;1(1):126.

[70] Delgado CL. Rising consumption of meat and milk in developing countries has created a new food revolution. J Nutr. 2003;133(11 Suppl 2):3907S-3910S. doi: 10.1093/jn/133.11.3907S 14672289

[71] Patel S, Patel J, Patel A. Gelani R. Role of women gender in livestock sector: A review, Journal of Livestock Science. 2016;7:92–6. http://www.wikigender.org

[72] Ali J. Livestock research for rural development. Livestock Research for Rural Development. 2007;19(2).

[73] Randolph TF, Schelling E, Grace D, Nicholson CF, Leroy JL, Cole DC, et al. Invited review: Role of livestock in human nutrition and health for poverty reduction in developing countries. J Anim Sci. 2007;85(11):2788–800. doi: 10.2527/jas.2007-0467 17911229

[74] Ababa A. Food processing & nutritional science determinants of dietary diversity consumption and nutritional status of pregnant women attending armed forces comprehensive specialized. Food Processing & Nutritional Science. 2020;1.

[75] Kiboi W, Kimiywe J, Chege P. Determinants of dietary diversity among pregnant women in Laikipia County, Kenya: a cross-sectional study. BMC Nutr. 2017;3(1). doi: 10.1186/s40795-017-0126-6

[76] Soharwardi MA, Ahmad TI. Dimensions and Determinants of Women Empowerment in Developing Countries. IJSDP. 2020;15(6):957–64. doi: 10.18280/ijsdp.150620

[77] Rivera RL, Zhang Y, Wang Q, Maulding MK, Tooze JA, Wright BN, et al. Diet Quality and Associations with Food Security among Women Eligible for Indiana Supplemental Nutrition Assistance Program-Education. J Nutr. 2020;150(8):2191–8. doi: 10.1093/jn/nxaa171 32559278 PMC7690761

[78] Rahman K, Islam M. Nutritional Status and Food Security of Farm Households under Different Land Use Patterns in Bangladesh. Bangladesh J Nutrition. 2013;:49–64. doi: 10.3329/bjnut.v24i0.14036

[79] Mannaf M, Uddin MT. Socioeconomic factors influencing food security status of maize growing households in selected areas of Bogra district., Bangladesh Journal of Agricultural Economics 2012;35:155–164. [cited January 24, 2024]. https://www.cabdirect.org/cabdirect/abstract/20163096487

[80] Guo L, Gu L, Peng Y, Gao Y, Mei L, Kang Q, et al. Online media exposure and weight and fitness management app use correlate with disordered eating symptoms: evidence from the mainland of China. J Eat Disord. 2022;10(1):58. doi: 10.1186/s40337-022-00577-y 35468844 PMC9036716

[81] Rothstein JD, Klemm R, Niyeha D, Smith E, Nordhagen S. Assessing the challenges to women’s access and implementation of text messages for nutrition behaviour change in rural Tanzania. Public Health Nutr. 2021;24(6):1478–91. doi: 10.1017/S1368980020003742 33118901 PMC8025099

[82] Sylvester G. Use of mobile phones by the rural poor: Gender perspectives from selected Asian countries. Ottawa, ON, CA: IDRC. 2016.

[83] Tran MC, Labrique AB, Mehra S, Ali H, Shaikh S, Mitra M, et al. Analyzing the mobile “digital divide”: changing determinants of household phone ownership over time in rural bangladesh. JMIR Mhealth Uhealth. 2015;3(1):e24. doi: 10.2196/mhealth.3663 25720457 PMC4376098

[84] Shah N, Zaheer S, Safdar NF, Turk T, Hashmi S. Women’s awareness, knowledge, attitudes, and behaviours towards nutrition and health in Pakistan: Evaluation of kitchen gardens nutrition program. PLoS One. 2023;18(9):e0291245. doi: 10.1371/journal.pone.0291245 37708133 PMC10501633

[85] Uddin MN, Roy P, Rahman S, Al-Amin AQ, Wahaj Z. Factors affecting rural women’s knowledge on food and nutrition: a case of specific areas of rural Bangladesh. Environ Dev Sustain. 2023;26(6):15619–37. doi: 10.1007/s10668-023-03266-1

