# Supplementary material for: Factors affecting women’s nutritional security in rural Bangladesh: The role of livestock and other socioeconomic characteristics
Source: PLoS One. 2025 Nov 20;20(11):e0335146. doi: 10.1371/journal.pone.0335146 (PMC12633883; doi:10.1371/journal.pone.0335146)
Supplement: S1 Table — (DOCX) [file pone.0335146.s001.docx]

**S1 Table:** Descriptive statistics of different independent variables

| **Variables** | **Entire households (n=5604)** | | | **Households have livestock (n=2725)** | | | **Households don’t have livestock (n=2879)** | | |
| --- | --- | --- | --- | --- | --- | --- | --- | --- | --- |
|  | Mean ± Std. Dev. | Min, Max | Frequency (percentage) | Mean ± Std. Dev. | Min, Max | Frequency (percentage) | Mean ± Std. Dev. | Min, Max | Frequency (percentage) |
| Years of schooling of women | 1.95±3.81 | 0, 16 |  | 1.98±3.83 | 0, 16 |  | 1.91±3.80 | 0,16 |  |
| Household size | 4.13±1.74 | 1, 18 |  | 4.40±1.77 | 1, 18 |  | 3.87±1.68 | 1,17 |  |
| Monthly income | 5496.27±7251.03 | 0, 125000 |  | 5807.99±6787.33 | 0, 80000 |  | 5201.23±7653.61 | 0,125000 |  |
| Farm size | 9.10±10.83 | 0, 170 |  | 9.85±10.72 | 0, 122 |  | 8.40±10.88 | 0,170 |  |
| Have livestock |  |  |  |  |  |  |  |  |  |
| no |  |  | 2879 (51.37) |  |  |  |  |  |  |
| yes |  |  | 2725 (48.63) |  |  |  |  |  |  |
| Employment status |  |  |  |  |  |  |  |  |  |
| no |  |  | 886 (15.81) |  |  | 125 (4.59) |  |  | 760(26.40) |
| yes |  |  | 4719 (84.19) |  |  | 2600 (95.41) |  |  | 2119(73.60) |
| Own mobile phone |  |  |  |  |  |  |  |  |  |
| no |  |  | 2559 (45.66) |  |  | 1415 (51.93) |  |  | 1143(39.70) |
| yes |  |  | 3046 (54.34) |  |  | 1310 (48.07) |  |  | 1736(60.30) |
| Nutritional knowledge |  |  |  |  |  |  |  |  |  |
| poor to fair |  |  | 5253 (93.72) |  |  | 2556 (93.8) |  |  | 2696(93.64) |
| good |  |  | 352 (6.28) |  |  | 169 (6.2) |  |  | 183(6.36) |
| Session with doctor |  |  |  |  |  |  |  |  |  |
| no |  |  | 4951 (88.33) |  |  | 2373 (87.08) |  |  | 2577(89.51) |
| yes |  |  | 654 (11.67) |  |  | 352 (12.92) |  |  | 302(10.49) |

Note: The methodology outlines three categories for classifying women's nutritional knowledge. However, due to the very low proportion of the “poor” category, it has been merged with the `fair` category and labelled as `poor to fair' nutritional knowledge among women.
